# Serious Games in Nursing Education: Scoping Review of Applications, Effectiveness, and Future Directions

**DOI:** 10.2196/86092

**Published:** 2026-06-11

**Authors:** Fenglin Shao, Zhihao Han, Qin Su, Yong Fang

**Affiliations:** 1 Respiratory Medicine Department, First Affiliated Hospital Zhejiang University, Hangzhou, China; 2 School of Nursing, Zhejiang Chinese Medical University, Hangzhou, China; 3 School of Nursing, Second Affiliated Hospital of Zhejiang Chinese Medical University, Hangzhou, China; 4 School of Nursing, Changsha Medical University, Leifeng Road, Changsha, Hunan, 410219, China, 86 17858565080

**Keywords:** nursing education, nursing students, serious games, game-based learning, simulation-based education, gamification, scoping review

## Abstract

**Background:**

Serious games (SGs) have emerged as a promising tool in nursing education, providing interactive learning environments for clinical simulation, skill development, and feedback. These games enhance knowledge, clinical reasoning, and psychomotor skills. However, evidence on their effectiveness is dispersed across various platforms and outcome measures, making it difficult to derive clear guidelines for their integration into nursing curricula.

**Objective:**

This scoping review aimed to systematically identify and map existing evidence on the use of SGs in nursing education, analyze game characteristics, and identify critical gaps to inform future research and practice development.

**Methods:**

This scoping review followed the JBI framework and the PRISMA-ScR checklist. Nine databases (PubMed, Web of Science, Embase, CINAHL, the Cochrane Library, CBM, Wanfang Data, CNKI, and VIP) were searched from inception to January 15, 2026. Eligible studies were those reporting on original research on SGs in nursing education. Two reviewers screened titles, abstracts, and full texts. Risk of bias was assessed using a standardized checklist. The extracted data encompassed study characteristics, study design, participant information, sample sizes, application context, teaching content, and SG characteristics. Data were extracted and synthesized with descriptive statistics and content analysis. An evidence gap map was created to show the study distribution across course categories and outcome domains.

**Results:**

We screened 6078 records and included 24 studies. Publications were from 2021 to 2025 (n=13, 54%), with the majority conducted in Europe (n=13, 54%). Quasi-experimental designs (n=10, 42%) and randomized controlled trials (n=8, 33%) were predominant. SGs were mainly used in fundamental or skills training and adult nursing courses. Scenario-based decision points (n=20, 83%) and points, badges, or leaderboards (n=20, 83%) were the most common game mechanics, while progression or unlocking and collaborative elements were less frequent. Outcomes most often assessed were knowledge (n=16, 67%), skills (n=10, 42%), and engagement or usability (n=13, 54%). Objective use metrics were rarely reported (n=1, 4%), indicating limited data on in-platform learning behaviors. Most SGs were delivered as digital non–virtual reality applications or computer-based simulation games. Follow-up assessment beyond immediate postintervention outcomes was infrequent. An evidence gap map showed studies concentrated in skills-based training and adult nursing, with fewer studies in maternity or neonatal nursing, critical care, and foundational sciences.

**Conclusions:**

This review extends earlier work on SGs in nursing education by mapping evidence across curricular areas, intervention reporting, and outcome assessment, rather than focusing mainly on effectiveness or specific formats. It shows where evidence is concentrated and where important gaps remain, particularly in underrepresented course areas, intervention descriptions, follow-up assessments, and objective use data. These findings provide a clearer picture of the evidence base and can inform curriculum planning; the use of SGs in skills-based and clinical training; and future decisions about their design, implementation, and evaluation.

## Introduction

### Serious Games in Nursing Education

Nursing education faces the growing challenge of delivering competency-based training at scale while maintaining learner engagement. This is especially challenging in nursing, where opportunities for clinical exposure and repeated skills practice are often limited due to patient safety concerns and restricted training resources [1]. Limited clinical placement capacity and variability in supervision can further restrict opportunities for standardized practice and skills assessment. Digital learning technologies can provide interactive, repeatable training beyond traditional lectures and demonstrations [2,3]. Alongside advances in commercial video game technologies, serious games (SGs) have emerged as educational applications that integrate engagement with explicit instructional aims [4]. SGs typically incorporate structured challenges, timely feedback, and progression or reward systems intended to support competency development [5]. Through scenario-based tasks and iterative problem-solving, SGs can provide standardized, low-risk environments for rehearsal and skills consolidation and have been associated with improvements in learning outcomes, including knowledge acquisition and skill performance [6].

Within nursing education, the use of SGs has expanded as programs seek scalable approaches aligned with competency-based curricula [7,8]. SGs have been applied across learning domains, including knowledge, psychomotor skills, clinical reasoning, teamwork, and communication [7,8]. SGs may also support self-regulated learning by clarifying goals, enabling frequent performance feedback, and facilitating repeated practice [9]. In this review, SGs are defined operationally as game-based learning interventions designed for educational purposes that include identifiable game mechanisms such as rules, structured challenges, feedback, and progression or reward systems [10,11]. SGs were distinguished from gamification-only activities by requiring a rule-based game structure with progression aligned to learning objectives, rather than the isolated addition of points or leaderboards to conventional teaching [11].

SGs are conceptually related to gamification, defined as the application of game design elements in nongame contexts to support motivation and behavior change [10,11]. Gamification is commonly described in terms of learner dynamics, alignment with curricular objectives, and mechanics such as rules, feedback, and progression structures [11]. In practice, these elements are often operationalized through points, badges, leaderboards, and milestone schedules that structure engagement and perceived progress [12]. In this review, classroom-based gamified activities delivered via web platforms or experiential formats were included when implemented as structured learning games with explicit educational objectives and reported outcomes.

Existing systematic reviews and meta-analyses of SGs or game-based interventions in nursing education have predominantly examined effectiveness under controlled trial conditions. Meta-analyses of randomized controlled studies report that SGs may improve learning outcomes, most frequently assessed using knowledge tests and performance-based skills measures [13-15]. A systematic review and meta-analysis focusing on knowledge outcomes further supports the potential of game-based interventions to enhance nursing students’ knowledge [16]. In addition, a systematic review and meta-analysis examining feedback elements suggests that feedback-related design mechanics may contribute to learning gains, underscoring the relevance of design components for educational impact [17]. Collectively, these syntheses summarize and, in some cases, quantify the extent to which SGs are associated with improvements in predefined outcomes and, in some cases, the contribution of specific design mechanics [18]. However, these reviews largely prioritize randomized controlled trials and short-term postintervention outcomes, with limited attention to implementation context, competency-domain coverage, and heterogeneity in outcome measurement.

### Research Gap and Objectives

Nonetheless, important gaps remain that constrain interpretation and implementation. First, effectiveness-focused syntheses offer limited coverage of quasi-experimental, qualitative, and mixed methods evidence that can inform feasibility, acceptability, contextual fit, and plausible mechanisms in nursing education [19]. Second, there is limited characterization of how outcomes are operationalized across studies, including the instruments used and the alignment between outcome selection, targeted competencies, and learning context [20]. Third, the intervention landscape remains insufficiently characterized with respect to SG types, instructional topics and settings, delivery modalities, and the distribution of evidence across these categories [19]. These limitations reduce comparability across studies and make it difficult to translate effectiveness estimates into actionable guidance for curriculum design and evaluation [19].

Accordingly, this study follows the JBI scoping review framework to systematically identify, map, and synthesize the evidence on SGs in nursing education. This review addresses three questions: (1) What SGs have been developed for nursing education or practice? (2) In which teaching or clinical contexts and competency domains have these SGs been implemented? and (3) How have their effectiveness and related outcomes been evaluated and reported?

## Methods

### Study Design

This study followed a scoping review approach in accordance with the JBI framework for scoping reviews [21]. The review process included defining the objectives and questions; developing the inclusion criteria; planning the search, selection, data extraction, analysis, and presentation methods; conducting the search; selecting and extracting the evidence; analyzing the evidence; presenting the results; and summarizing the findings in line with the purpose of the review [21]. The findings are reported in accordance with the PRISMA-ScR (Preferred Reporting Items for Systematic Reviews and Meta-Analyses Extension for Scoping Reviews) checklist (Checklist 1) [22].

### Protocol and Registration

No registration was undertaken for this scoping review; the protocol is provided for transparency (Multimedia Appendix 1).

### Search Strategy

The literature search was developed by the review team and combined controlled vocabulary terms and free-text keywords related to SGs and nursing education. English-language databases included PubMed, Web of Science, Embase, CINAHL, and the Cochrane Library. Chinese-language databases included China Biology Medicine, Wanfang Data, China National Knowledge Infrastructure, and VIP. These databases were initially searched from database inception to June 28, 2024, and the searches were rerun on January 28, 2026, to identify newly published records. Each database was searched individually, and no simultaneous multidatabase searching on a single platform was used. No additional search methods were used, including study registry searching, manual searching of reference lists, forward citation tracking, website browsing, conference proceedings searching, or author or expert contact. No published search filters were used or adapted in this review. The search strategies were reviewed by a library specialist to improve completeness and accuracy before the searches were conducted. The full search strategies for each database are provided in Multimedia Appendix 2.

### Eligibility Criteria

To ensure the selection of relevant studies, specific inclusion and exclusion criteria were established based on the scope of this review. The eligibility criteria are shown in Textbox 1.

Textbox 1. **Study eligibility criteria.**

**Inclusion criteria**

- Participants: undergraduate or graduate nursing students (including nursing interns or trainees when clearly identified as nursing learners).
- Concept: serious game (SG)–based teaching and learning interventions (digital, offline, or hybrid or blended formats) with an explicit educational or training purpose.
- Context: nursing education activities (eg, coursework or lectures, skills training, simulation, or clinical skills learning). Interventions were classified as SGs only if they included an explicit game structure (rules with a defined goal or end point), a trackable progression system (eg, multiround missions or levels with unlocking), and performance feedback tied to the intended learning objectives; otherwise, they were coded as gamification only and excluded.
- Article type: original study, journal article, or conference paper.
- Language: English or Chinese.

**Exclusion criteria**

- Secondary evidence syntheses (eg, reviews or meta-analyses) and nonempirical publications (eg, protocols, guidelines, expert opinions, editorials or commentaries, letters, or policy documents).
- Studies not meeting the population, concept, and context definitions.
- Duplicate reports of the same study or studies for which the full text was not available.

### Selection of Sources of Evidence

#### Title and Abstract Screening

The retrieved records were first deduplicated using EndNote. Two reviewers (QS and ZH) independently screened the titles and abstracts of all remaining records to identify studies that potentially met the inclusion criteria.

#### Full-Text Screening and Eligibility Assessment

Following title and abstract screening, the same 2 reviewers (QS and ZH) independently conducted a full-text assessment of potentially eligible studies to determine final eligibility. Discrepancies were resolved through discussion and cross-checking; if consensus could not be reached, a third reviewer (YF) provided adjudication.

#### Final Study Selection

Studies that met the eligibility criteria were included in the final review. The overall selection process is presented in a PRISMA (Preferred Reporting Items for Systematic Reviews and Meta-Analyses) flow diagram.

#### Data Charting Process and Data Items

Data extraction was independently performed by 2 reviewers using a piloted, standardized form developed by the review team. The form was designed in line with the review objectives and eligibility criteria to capture bibliographic details (country, authors, year), study design, participant information, sample sizes (for context), application context (course or setting), intervention measures, basic design info, intervention duration, teaching content, and SG characteristics, including modality or platform, delivery format, brief game description, game design (standardized), key game design features (mechanics), and evaluation indicators. For data charting and comparison across studies, the extracted information was further organized into broader categories, including study characteristics, educational context, intervention characteristics, game design features, and outcome assessment. Disagreements were resolved through discussion with a third reviewer.

#### Synthesis of Results

Descriptive statistics (frequencies or percentages) and narrative synthesis were used to summarize the data; no meta-analysis was conducted. Study characteristics such as country, year of publication, study design, and sample size were summarized to provide an overview of the distribution of the included studies. Educational context data were categorized by nursing course type and teaching content to highlight the areas where SGs were most commonly applied. Intervention characteristics, including modality or platform, delivery format, intervention duration, brief game descriptions, standardized game design, and key game mechanics, were summarized across the studies. Evaluation indicators were grouped into broader outcome domains such as knowledge, skills, attitudes, and engagement or usability to facilitate cross-study comparison. The extracted data were presented in descriptive tables to ensure transparency, and a narrative thematic synthesis was conducted to identify patterns across different game types, educational contexts, and outcome domains. Additionally, an evidence gap map was developed to visualize the distribution of studies across nursing course categories and outcome domains.

#### Critical Appraisal

We appraised methodological quality using JBI critical appraisal tools tailored to study design. Two reviewers independently assessed each included study using item-level ratings of yes, no, unclear, or not applicable; disagreements were resolved by discussion, with third-reviewer adjudication when necessary. Consistent with scoping review methodology, studies were not excluded based on appraisal; findings were used to contextualize confidence in the evidence and to inform interpretation of results and evidence gaps.

## Results

### Study Selection

**Figure 1.**
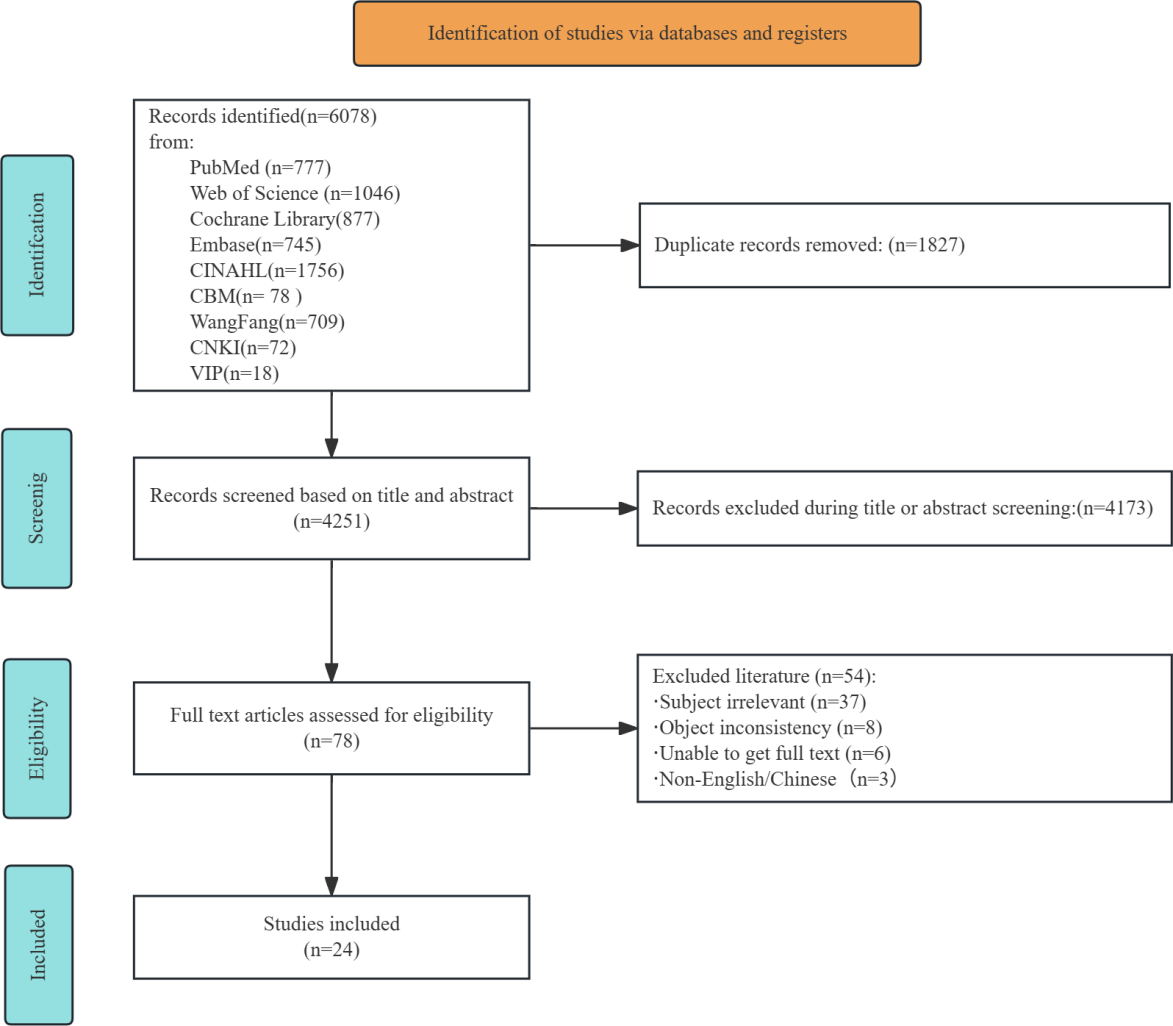
PRISMA flow diagram of study selection.

The initial search yielded 6078 records, of which 1827 (30.06%) were duplicates and 4173 (68.65%) were excluded after screening titles and abstracts. Of the remaining 78 full-text articles assessed for eligibility, 24 studies [23-46] met the inclusion criteria. The PRISMA-ScR flowchart (Figure 1) provides further details.

### Characteristics of the Included Studies

Of the 24 included studies, publication dates clustered from 2021 to 2025 (n=13, 54%), followed by 2017 to 2020 (n=7, 29%) and 2012 to 2016 (n=4, 17%). Studies were predominantly from Europe (n=13, 54%), with additional studies from Asia (n=5, 21%) and North America (n=4, 17%); Oceania and South America were each represented by 1 study (n=1, 4%). Quasi-experimental designs were most common (n=10, 42%), followed by randomized controlled trials (n=8, 33%), mixed methods studies (n=4, 17%), and qualitative studies (n=2, 8%; Table 1).

**Table 1.** Characteristics of included studies on serious games in nursing education (N=24).

Characteristic	Studies, n (%)
Publication year	
2012‐2016	4 (17)
2017‐2020	7 (29)
2021‐2025	13 (54)
Country or region (continent)	
Europe	13 (54)
Asia	5 (21)
North America	4 (17)
Oceania	1 (4)
South America	1 (4)
Study design	
Randomized controlled trial	8 (33)
Quasi-experimental	10 (42)
Mixed methods	4 (17)
Qualitative	2 (8)

### Results of Individual Sources of Evidence

The extracted data from the included studies are presented in Multimedia Appendix 3, including study characteristics, educational context, intervention details, SG characteristics, and evaluation indicators, and form the basis for the analyses presented below.

### Synthesis of Results

#### Delivery Format and Key Game Design Mechanics of SGs

**Figure 2.**
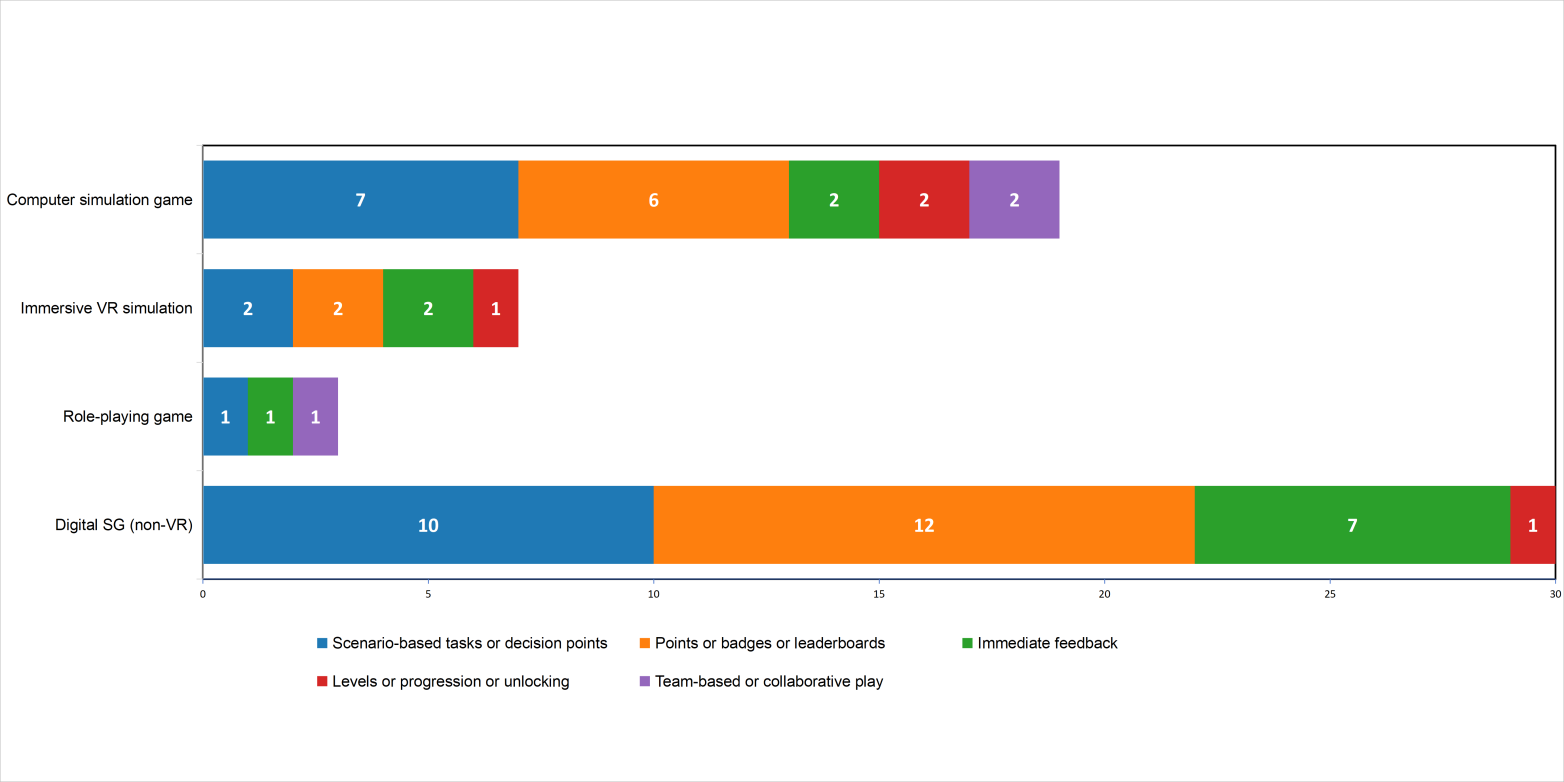
Distribution of key game design mechanics by serious game (SG) delivery format in nursing education studies. VR: virtual reality.

Each study was assigned to 1 primary delivery format based on the main platform reported. Digital SGs delivered via non–virtual reality (VR) platforms were most common (14/24, 58%), followed by computer simulation games (n=74, 29%), immersive VR simulations (n=2, 8%), and role-playing games (n=1, 4%). Scenario-based tasks and decision points were reported in 20 (83%) studies, as were points, badges, and/or leaderboards (n=20, 83%). Immediate feedback was reported in 12 (50%) studies, whereas levels or progression or unlocking (n=4, 17%) and team-based or collaborative play (n=3, 13%) were less frequently reported (Table 2). Figure 2 provides a visual representation of the data summarized in Table 2, illustrating how the reported game design mechanics are distributed across delivery formats.

**Table 2.** Delivery format and key game design mechanics of serious games used in nursing education (N=24)^a^.

Characteristic	Studies, n (%)
Delivery format	
Computer simulation game	7 (29)
Immersive VR^b^ simulation	2 (8)
Role-playing game	1 (4)
Digital SG^c^ (non-VR)	14 (58)
Key game design mechanics	
Scenario-based tasks or decision points	20 (83)
Points, badges, and/or leaderboards	20 (83)
Immediate feedback	12 (50)
Levels or progression or unlocking	4 (17)
Team-based or collaborative play	3 (13)

a One study may contribute to multiple design mechanics; scenario-based tasks or decision points were coded when studies explicitly described case-based scenarios and learner choices affecting task progression or responses; points, badges, and/or leaderboards indicate reporting of 1 or more of these mechanics; and levels or progression or unlocking indicate an explicit staged progression or unlocking pathway.

b VR: virtual reality.

c SG: serious game.

#### Nursing Course Categories and Supported Topics

SG-supported learning was predominantly used in skills-based courses, with additional implementation observed in specialty nursing areas. Across the 24 included studies, SG-supported learning activities were most commonly implemented in fundamentals and skills training courses (7/24, 29%), followed by adult nursing and medical-surgical courses (n=6, 25%). Fundamentals and skills training applications focused on core procedural competencies such as cardiopulmonary resuscitation and life support, safe transfusion management, and intramuscular injection skills training. Adult nursing applications covered medical-surgical topics including electrocardiogram interpretation, chronic disease care, symptom recognition and management in acute heart failure, tracheostomy care, early warning and recognition of postoperative hip bleeding, and diabetic ketoacidosis care. Pediatric nursing and community or primary care nursing were each represented by 3 (13%) studies addressing pediatric assessment or care topics and community-oriented competencies such as nurse-patient communication and environmental risk assessment. Critical care nursing was represented by 2 (8%) studies, while maternity or neonatal nursing, emergency care, and foundational sciences were each represented by 1 (4%) study. Table 3 summarizes the course categories and topics addressed.

**Table 3.** Course categories and nursing courses or topics supported by serious games (SGs; N=24)^a^.

Course category	Studies, n (%)	Nursing courses or topics supported by SGs
Fundamental or skills training	7 (29)	Cardiopulmonary resuscitation training Basic and advanced life support training Safe transfusion management Intramuscular injection skills training
Adult nursing (medical-surgical)	6 (25)	Electrocardiogram interpretation Care of patients with chronic diseases Identification and care of acute heart failure symptoms Nursing care of patients with tracheostomies Early warning and recognition of postoperative hip bleeding Nursing care of patients with diabetic ketoacidosis
Pediatric nursing	3 (13)	Clinical assessment of respiratory problems in premature infants Nursing care of children after appendectomy Teaching pediatric respiratory system knowledge
Maternity or neonatal nursing	1 (4)	Neonatal cardiopulmonary resuscitation training
Community or primary care nursing	3 (13)	Nurse-patient communication Community and public health nursing competencies training Risk assessment of the living environments of older adults
Emergency care	1 (4)	Team collaboration in the emergency department
Critical care nursing	2 (8)	Assessment of critical care nursing skills Analysis of pneumonia complicated by sepsis
Foundational sciences	1 (4)	Pathophysiology teaching

a Each study was assigned to 1 primary course category based on the main educational setting or course described by the authors; course labels were harmonized across studies into common nursing curriculum modules.

#### Evidence Gap Map Across Course Categories and Outcome Domains

**Figure 3.**
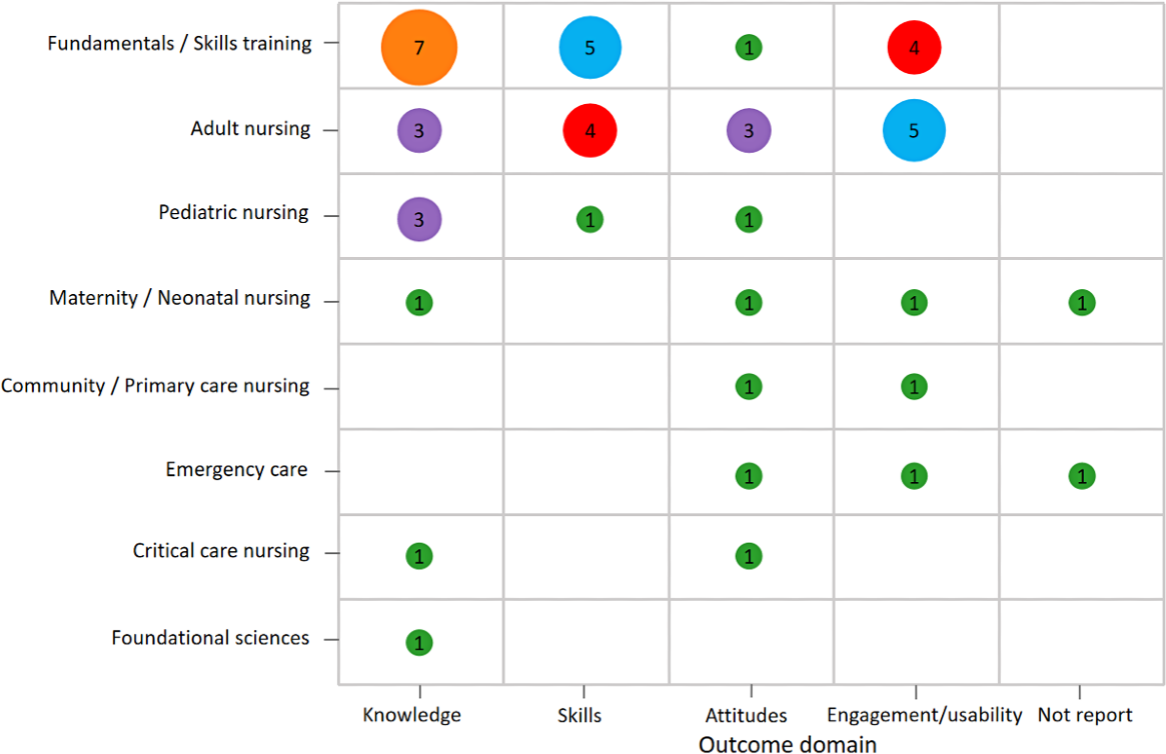
Evidence gap map of serious game studies in nursing education by course category and outcome domain.

Of the 24 included studies, outcome domains and measurement approaches were unevenly distributed, revealing several clear evidence gaps. Knowledge was the most frequently assessed outcome, reported in 16 (67%) studies using tests. Skills were assessed in 10 (42%) studies, including 3 (13%) studies using tests and 7 (29%) studies using checklists. Attitudes were measured in 8 (33%) studies using scales, and engagement or usability in 13 (54%) studies, also mainly using scales. By contrast, use metrics were reported in only 1 (4%) study, limited to backend interaction data such as frequency of use and time spent, indicating a substantial lack of evidence on how learners actually use SGs in practice. A total of 2 (8%) qualitative studies did not report specific quantitative outcome measures, further suggesting limited evidence on learners’ experiences outside narrative accounts (Table 4). The evidence gap map (Figure 3) showed that existing studies were concentrated in fundamentals or skills training and adult nursing, whereas maternity or neonatal nursing, emergency care, critical care nursing, and foundational sciences were represented by very few studies. Across outcome domains, the evidence was heavily weighted toward knowledge and, to a lesser extent, skills and engagement or usability, while attitudes, use patterns, and qualitative experience-related outcomes were comparatively sparse. Overall, the main evidence gaps lay in underrepresented course categories and in the limited assessment of implementation-related and experience-related outcomes beyond knowledge-based evaluation.

**Table 4.** Outcome domains, measurement types, and corresponding studies across included studies (N=24)^a^.

Outcome domain	Measurement type	Studies, n (%)	Included studies
Knowledge	Test	16 (67)	[29-36,38-42,44-46]
Skills	Test	3 (13)	[27,32,37]
	Checklist	7 (29)	[29,33-36,44,45]
Attitudes	Scale	8 (33)	[23,28,31,32,40,41,43,45]
	Use metrics	1 (4)	[30]
Engagement or usability	Scale	13 (54)	[23,24,27,28,31-33,37,39-41,45,46]
Not reported	—^b^	2 (8)	[25,26]

a Use metrics refer to backend use data of user interaction or engagement (eg, frequency of use and time spent). Not reported means that studies did not report specific outcome measurements due to their qualitative design.

b Not applicable.

#### Critical Appraisal

Methodological quality was assessed using design-appropriate JBI critical appraisal tools and the mixed methods appraisal tool (Multimedia Appendix 4). Overall, of the 24 studies, 19 met at least 75% of appraisal criteria, meaning they satisfied three-quarters or more of the applicable checklist items for their study design, with relatively few items rated as “no” or “unclear.” By study design, 7 out of 8 randomized controlled trials and 8 out of 10 quasi-experimental studies met at least 75% of criteria, compared with 1 out of 2 qualitative studies and 3 out of 4 mixed methods studies. Across designs, the most frequent limitations or unclear reporting concerned internal validity items and reporting transparency, which may constrain confidence in longer-term outcomes and implementation-related inferences.

## Discussion

### Principal Findings

This scoping review mapped the characteristics, curricular distribution, and outcome evaluation of SGs in nursing education and identified several important gaps in the existing evidence base. The literature was concentrated in practice-oriented areas, particularly skills training and adult nursing, and most commonly evaluated knowledge, skills, and learner-reported engagement or usability. By contrast, instructional rationale, implementation context, objective use data, and outcomes beyond the immediate postintervention period were infrequently reported. These findings suggest that SGs are currently used primarily as supplementary tools for practice-oriented learning in nursing education, while the uneven distribution of evidence and limited reporting constrain more robust inferences about their educational function, contextual suitability, and broader implementation.

Geographic concentration of the literature was another notable pattern in this review. One plausible explanation is that the development and evaluation of SGs require resources that are unevenly distributed across settings, such as reliable connectivity, access to appropriate devices, and technical support for deployment and data management [47-49]. Institutional capacity may also play a role in determining whether SGs can be integrated into teaching and assessment workflows and evaluated with sufficient rigor for publication [50]. Although this review was not designed to test these explanations directly, the geographic concentration of studies in better-resourced contexts suggests that nursing programs with fewer resources may be underrepresented in the published literature. This has implications for both the generalizability and equity of the findings and emphasizes the need for clearer reporting of implementation conditions, resource barriers, and local adaptations.

A recurring issue in the literature was that studies often described game features more clearly than the underlying educational logic. While many studies reported visible features such as scenario-based decision points and reward structures, fewer clearly articulated the instructional rationale behind these features or explained how feedback and progression were designed to support learning [18]. This lack of clarity weakens the interpretation of findings. In educational terms, the value of SGs lies not just in their digital or game-based format but in whether they support repeated practice, provide timely feedback, and calibrate challenge to maintain learner engagement while enhancing competence [17]. These principles align with experiential learning and self-determination theories, which suggest that short-term improvements in motivation, self-efficacy, and persistence are achievable [51]. However, the studies included in this review rarely specified these mechanisms in testable ways. As a result, it is difficult to determine which components of SG design are most effective in driving learning gains. Future research would benefit from moving beyond platform labels and providing more detailed descriptions of mechanics, feedback processes, progression rules, and the pedagogical assumptions linking these features to learning outcomes. This is particularly important given that different platforms (eg, mobile, web, and VR) may be more suitable for different educational goals: low-cost web or mobile formats may be more feasible in resource-limited settings, while immersive VR may be better suited for psychomotor training or spatial interaction when time, technical support, and usability allow [52,53].

The curricular pattern identified in this review also revealed significant trends. SG applications were predominantly clustered in skills training and adult nursing, which aligns with learning goals that are easier to operationalize through rules, cues, branching decisions, and feedback loops [18,19]. These game mechanics are well suited to practice-based learning, providing opportunities for repetition and error recovery [18,19]. In contrast, theory-heavy content requires more explicit conceptual scaffolding and progression structures that support abstraction, integration, and reflective reasoning [54]. Without such scaffolding, theoretical content risks being reduced to quiz-like gamification rather than facilitating deeper learning [18]. Additionally, the availability of commercially available products and off-the-shelf training modules may have influenced the prevalence of procedural training, as skill acquisition is easier to measure than conceptual understanding [55]. These patterns suggest that SGs are most effective when they offer educationally distinctive features such as safe repetition, exposure to rare or high-risk situations, or dynamic decision-making environments, rather than simply replicating traditional lecture content or assessments in a game format [42]. However, there is considerable potential to expand SG applications into more complex competencies such as clinical reasoning, communication, interprofessional collaboration, and patient safety habits [24,39]. Team-based simulation research supports the potential for improving teamwork and leadership in dynamic care environments, but these outcomes will require mechanics that foster coordination, communication, and shared situational awareness [56].

The way outcomes were measured also shaped the conclusions that could be drawn from the studies. Most studies relied on knowledge tests or learner-reported experiences, which are common in short-course evaluations [20]. While these measures provide useful insights into immediate educational effects, they do not capture sustained learning, transfer to clinical performance, or the mechanisms driving these effects [39]. This limitation is particularly evident in studies that lacked standardized measurement tools or sufficient follow-up, raising concerns about novelty effects or short-term gains. The predominance of quasi-experimental designs and small sample sizes further complicates the interpretation of findings [32,46]. A major gap in the literature is the lack of objective use data such as time on task, frequency of play, progression, or error patterns [57,58]. Without these indicators, it is difficult to explore dose-response relationships, evaluate which aspects of gameplay are most impactful, or understand how learners engage with SGs [59]. This limitation is critical, as the effectiveness of SGs relies on learners’ interaction with the game, not just the design itself. When properly captured, gameplay data could provide valuable insights into decision-making processes, feedback uptake, and error recovery; however, this potential remains largely untapped.

These patterns highlight several priorities for future research. Development, implementation, and evaluation of SGs should be treated as interconnected tasks rather than separate phases. SGs are unlikely to produce meaningful results if learning objectives are not clearly defined, usability is not sufficiently tested, or delivery conditions are misaligned with the curriculum [19]. A more robust approach would begin with clear articulation of learning goals and contextual constraints, followed by an explicit linking of those goals to game mechanics, feedback logic, and progression design. Iterative user-centered refinement is also essential, particularly where usability or acceptability may influence uptake. Evaluation should extend beyond short-term effectiveness, incorporating indicators such as acceptability, satisfaction, perceived usefulness, usability, adoption, and engagement [60,61]. Objective use metrics are particularly important to assess dose, fidelity, and mechanisms. Where feasible, longer-term outcomes such as retention, transfer to practice, cost-effectiveness, and equity-related effects should be included to better inform adoption decisions across diverse nursing programs [62,63].

### Limitations

This review has several limitations. First, only studies published in Chinese or English were included, and relevant evidence in other languages may have been missed. Second, gray literature and unpublished work were not included, and some SGs are privately developed with limited disclosure of development and evaluation details. Third, substantial heterogeneity in interventions and outcome measures, together with short follow-up and limited reporting of use data, constrained comparability and precluded quantitative synthesis. Finally, incomplete reporting of implementation context and reliance on self-report or nonstandardized measures limit reproducibility and warrant caution when interpreting improved learner experience as competence gains.

### Conclusions

This review extends earlier work on SGs in nursing education by mapping the evidence across curricular areas, intervention reporting, and outcome assessment, rather than focusing mainly on effectiveness or specific formats. The available evidence is concentrated in practice-oriented topics, while important gaps remain in underrepresented course areas, intervention description, follow-up assessment, and objective use data. Together, these findings offer a clearer picture of how SGs are being studied in nursing education and where stronger evidence is still needed. They may inform curriculum planning, support more targeted use of SGs in skills-based and clinical training, and guide decisions about design, implementation, and evaluation in real educational settings. Future studies should describe interventions more clearly and assess implementation and longer-term outcomes more consistently.

## Supplementary material

10.2196/86092 Multimedia Appendix 1 Protocol for the scoping review.

10.2196/86092 Multimedia Appendix 2 Search strategies.

10.2196/86092 Multimedia Appendix 3 Data extraction results.

10.2196/86092 Multimedia Appendix 4 Critical appraisal.

10.2196/86092 Checklist 1 PRISMA-ScR checklist.

## Abbreviations

PRISMA: Preferred Reporting Items for Systematic Reviews and Meta-Analyses
PRISMA-ScR: Preferred Reporting Items for Systematic Reviews and Meta-Analyses Extension for Scoping Reviews
SG: serious game
VR: virtual reality

## References

[1] Alkhaledi NG, Alabdalhai SA, Awaji NY (2024). Utilizing competency-based education to evaluate the research skills of nursing students: a systematic review and meta-analysis. Cureus.

[2] Ota Y, Aikawa G, Nishimura A, Kawashima T, Imanaka R, Sakuramoto H (2024). Effects of educational methods using extended reality on pre-registration nursing students’ knowledge, skill, confidence, and satisfaction: a systematic review and meta-analysis. Nurse Educ Today.

[3] O’Connor S, Wang Y, Cooke S (2023). Designing and delivering digital learning (e-Learning) interventions in nursing and midwifery education: a systematic review of theories. Nurse Educ Pract.

[4] Abd-Alrazaq A, Abuelezz I, Hassan A (2022). Artificial intelligence-driven serious games in health care: scoping review. JMIR Serious Games.

[5] López-Bouzas N, Del Moral-Pérez ME (2023). Gamified environments and serious games for students with autistic spectrum disorder: review of research. Rev J Autism Dev Disord.

[6] Gentry SV, Gauthier A, L’Estrade Ehrstrom B (2019). Serious gaming and gamification education in health professions: systematic review. J Med Internet Res.

[7] Calik A, Kapucu S (2024). Comparative effectiveness of developed serious game versus standardized patients’ simulation in nursing education. Games Health J.

[8] Demircan B, Kıyak Y, Kaya H (2024). The effectiveness of serious games in nursing education: a meta-analysis of randomized controlled studies. Nurse Educ Today.

[9] Johnsen HM, Fossum M, Vivekananda-Schmidt P, Fruhling A, Slettebø Å (2018). Developing a serious game for nurse education. J Gerontol Nurs.

[10] Warsinsky S, Schmidt-Kraepelin M, Rank S, Thiebes S, Sunyaev A (2021). Conceptual ambiguity surrounding gamification and serious games in health care: literature review and development of game-based intervention reporting guidelines (GAMING). J Med Internet Res.

[11] Khaldi A, Bouzidi R, Nader F (2023). Gamification of e-learning in higher education: a systematic literature review. Smart Learn Environ.

[12] van Gaalen AE, Brouwer J, Schönrock-Adema J, Bouwkamp-Timmer T, Jaarsma AD, Georgiadis JR (2021). Gamification of health professions education: a systematic review. Adv Health Sci Educ Theory Pract.

[13] Min A, Min H, Kim S (2022). Effectiveness of serious games in nurse education: a systematic review. Nurse Educ Today.

[14] Thangavelu DP, Tan AJ, Cant R, Chua WL, Liaw SY (2022). Digital serious games in developing nursing clinical competence: a systematic review and meta-analysis. Nurse Educ Today.

[15] Cheng P, Huang Y, Yang P (2024). The effects of serious games on cardiopulmonary resuscitation training and education: systematic review with meta-analysis of randomized controlled trials. JMIR Serious Games.

[16] Celik F, Turan R, Bektas H (2025). The effect of game-based interventions on the nursing students’ level of knowledge: a systematic review and meta-analysis of randomized controlled trials. Nurse Educ Today.

[17] Liu Z, Yu R, Yao X, Yan Q (2025). The impact of feedback elements in serious games on nursing learning outcomes: a systematic review and meta-analysis. Nurse Educ Today.

[18] Aster A, Laupichler MC, Zimmer S, Raupach T (2024). Game design elements of serious games in the education of medical and healthcare professions: a mixed-methods systematic review of underlying theories and teaching effectiveness. Adv Health Sci Educ Theory Pract.

[19] Chatzea VE, Logothetis I, Kalogiannakis M, Rovithis M, Vidakis N (2025). Digital serious games for undergraduate nursing education: a review of serious games key design characteristics and gamification elements. Information.

[20] Lee M, Shin S, Lee M, Hong E (2024). Educational outcomes of digital serious games in nursing education: a systematic review and meta-analysis of randomized controlled trials. BMC Med Educ.

[21] Peters MD, Marnie C, Tricco AC (2020). Updated methodological guidance for the conduct of scoping reviews. JBI Evid Synth.

[22] Tricco AC, Lillie E, Zarin W (2018). PRISMA extension for scoping reviews (PRISMA-ScR): checklist and explanation. Ann Intern Med.

[23] Calik A, Kapucu S (2022). The effect of serious games for nursing students in clinical decision-making process: a pilot randomized controlled trial. Games Health J.

[24] Hara CY, Goes F, Camargo RA, Fonseca LM, Aredes ND (2021). Design and evaluation of a 3D serious game for communication learning in nursing education. Nurse Educ Today.

[25] Roman P, Ruiz-Gonzalez C, Rodriguez-Arrastia M, Granero-Molina J, Fernández-Sola C, Hernández-Padilla JM (2022). A serious game for online-based objective structured clinical examination in nursing: a qualitative study. Nurse Educ Today.

[26] Volejnikova-Wenger S, Andersen P, Clarke KA (2021). Student nurses’ experience using a serious game to learn environmental hazard and safety assessment. Nurse Educ Today.

[27] Maheu-Cadotte MA, Dubé V, Lavoie P (2023). Development and contribution of a serious game to improve nursing students’ clinical reasoning in acute heart failure: a multimethod study. Comput Inform Nurs.

[28] Wong JY, Ko J, Nam S (2022). Virtual ER, a serious game for interprofessional education to enhance teamwork in medical and nursing undergraduates: development and evaluation study. JMIR Serious Games.

[29] Demirtas A, Basak T, Sahin G, Sonkaya MÇ (2022). The serious game and integrated simulator for cardiopulmonary resuscitation training in nursing students. Simul Gaming.

[30] Sarvan S, Efe E (2022). The effect of neonatal resuscitation training based on a serious game simulation method on nursing students’ knowledge, skills, satisfaction and self-confidence levels: a randomized controlled trial. Nurse Educ Today.

[31] Adhikari R, Kydonaki C, Lawrie J (2021). A mixed-methods feasibility study to assess the acceptability and applicability of immersive virtual reality sepsis game as an adjunct to nursing education. Nurse Educ Today.

[32] Chang CY, Kao CH, Hwang GJ, Lin FH (2020). From experiencing to critical thinking: a contextual game-based learning approach to improving nursing students’ performance in electrocardiogram training. Educ Technol Res Dev.

[33] Tan AJQ, Lee CCS, Lin PY (2017). Designing and evaluating the effectiveness of a serious game for safe administration of blood transfusion: a randomized controlled trial. Nurse Educ Today.

[34] Bayram SB, Caliskan N (2019). Effect of a game-based virtual reality phone application on tracheostomy care education for nursing students: a randomized controlled trial. Nurse Educ Today.

[35] Boada I, Rodriguez-Benitez A, Garcia-Gonzalez JM, Olivet J, Carreras V, Sbert M (2015). Using a serious game to complement CPR instruction in a nurse faculty. Comput Methods Programs Biomed.

[36] LeFlore JL, Anderson M, Zielke MA (2012). Can a virtual patient trainer teach student nurses how to save lives--teaching nursing students about pediatric respiratory diseases. Simul Healthc.

[37] Blanié A, Amorim MA, Benhamou D (2020). Comparative value of a simulation by gaming and a traditional teaching method to improve clinical reasoning skills necessary to detect patient deterioration: a randomized study in nursing students. BMC Med Educ.

[38] Fonseca LM, Aredes ND, Fernandes AM (2016). Computer and laboratory simulation in the teaching of neonatal nursing: innovation and impact on learning [Article in English, Portuguese, Spanish]. Rev Lat Am Enfermagem.

[39] Gutiérrez-Puertas L, García-Viola A, Márquez-Hernández VV, Garrido-Molina JM, Granados-Gámez G, Aguilera-Manrique G (2021). Guess it (SVUAL): an app designed to help nursing students acquire and retain knowledge about basic and advanced life support techniques. Nurse Educ Pract.

[40] Kang J, Suh EE (2018). Development and evaluation of “chronic illness care smartphone apps” on nursing students’ knowledge, self-efficacy, and learning experience. Comput Inform Nurs.

[41] Verkuyl M, Romaniuk D, Atack L, Mastrilli P (2017). Virtual gaming simulation for nursing education: an experiment. Clin Simul Nurs.

[42] Ignacio J, Chen HC (2020). The use of web-based classroom gaming to facilitate cognitive integration in undergraduate nursing students: a mixed methods study. Nurse Educ Pract.

[43] Hughes MJ (2025). Enhancing public health nursing competencies through game-based education. Nurse Educ.

[44] Fijačko N, Masterson Creber R, Metličar Š (2024). Effects of a serious smartphone game on nursing students’ theoretical knowledge and practical skills in adult basic life support: randomized wait list-controlled trial. JMIR Serious Games.

[45] Gokalp MG, Yucel SC, Yilmaz O (2025). The effect of game-based learning on the acquisition of intramuscular injection skills. Nurse Educ Pract.

[46] Cook NF, McAloon T, O’Neill P, Beggs R (2012). Impact of a web based interactive simulation game (PULSE) on nursing students’ experience and performance in life support training--a pilot study. Nurse Educ Today.

[47] Wang R, DeMaria S, Goldberg A, Katz D (2016). A systematic review of serious games in training health care professionals. Simul Healthc.

[48] Lie SS, Helle N, Sletteland NV, Vikman MD, Bonsaksen T (2023). Implementation of virtual reality in health professions education: scoping review. JMIR Med Educ.

[49] Marcu G, Ondersma SJ, Spiller AN, Broderick BM, Kadri R, Buis LR (2022). Barriers and considerations in the design and implementation of digital behavioral interventions: qualitative analysis. J Med Internet Res.

[50] MacKinnon K, Marcellus L, Rivers J, Gordon C, Ryan M, Butcher D (2017). Student and educator experiences of maternal-child simulation-based learning: a systematic review of qualitative evidence. JBI Database System Rev Implement Rep.

[51] Daoudi I (2022). Learning analytics for enhancing the usability of serious games in formal education: a systematic literature review and research agenda. Educ Inf Technol (Dordr).

[52] Armour T, Coffey E, Manias E, Redley B, Nicholson P (2025). Development of mobile educational applications designed for nurses: a narrative review. Nurse Educ Today.

[53] Ryan GV, Callaghan S, Rafferty A, Higgins MF, Mangina E, McAuliffe F (2022). Learning outcomes of immersive technologies in health care student education: systematic review of the literature. J Med Internet Res.

[54] Guth TA, Wolfe RM, Martinez O (2024). Assessment of clinical reasoning in undergraduate medical education: a pragmatic approach to programmatic assessment. Acad Med.

[55] Chan JY, Closser AH, Ngo V, Smith H, Liu AS, Ottmar E (2023). Examining shifts in conceptual knowledge, procedural knowledge and procedural flexibility in the context of two game‐based technologies. J Comput Assist Learn.

[56] Wang L, Zhao Q, Dong L (2024). The effectiveness of serious games on undergraduate nursing students’ knowledge and skills: a systematic review and meta-analysis. Nurse Educ Pract.

[57] Martinengo L, Ng MS, Ng TD (2024). Spaced digital education for health professionals: systematic review and meta-analysis. J Med Internet Res.

[58] Kim JH, Park H (2019). Effects of smartphone-based mobile learning in nursing education: a systematic review and meta-analysis. Asian Nurs Res (Korean Soc Nurs Sci).

[59] Kang J, Liu M, Qu W (2017). Using gameplay data to examine learning behavior patterns in a serious game. Comput Human Behav.

[60] Kwak M, Kim BJ, Chung JB (2024). Serious game development for public health: participatory design approach to COVID-19 quarantine policy education. JMIR Serious Games.

[61] Caldas OI, Mauledoux M, Aviles OF, Rodriguez-Guerrero C (2024). Breaking presence in immersive virtual reality toward behavioral and emotional engagement. Comput Methods Programs Biomed.

[62] Flato UA, Flato A, Martins I (2025). Enhancing equity in schoolchildren’s basic life support education in Brazil through serious games: cohort study. JMIR Serious Games.

[63] Allan R, McCann L, Johnson L, Dyson M, Ford J (2023). A systematic review of “equity-focused” game-based learning in the teaching of health staff. Public Health Pract (Oxf).

