# Supplementary material for: Serious Games in Nursing Education: Scoping Review of Applications, Effectiveness, and Future Directions
Source: JMIR Serious Games. 2026 Jun 11;14:e86092. doi: 10.2196/86092 (PMC13258065; doi:10.2196/86092)
Supplement: Multimedia Appendix 1 [file games-v14-e86092-s001.docx]

**Protocol**

**Application of Serious Games in Nursing Education – A Scoping Review**

Yong Fang; Qin Su; Zhihao Han; Chaoya Li; Chao Xu.

**1. Background and rationale**

Serious games and related digital game-based learning approaches are increasingly used in nursing education, yet the evidence remains heterogeneous across platforms, educational contexts, study designs, and outcome measures. This scoping review aims to map the extent, range, and nature of the available evidence, identify evidence clusters and gaps, and inform future research and educational implementation.

**2. Objectives**

To map applications of serious games in nursing education, summarize reported evaluation outcomes, identify evidence clusters and gaps, and appraise methodological quality using Joanna Briggs Institute critical appraisal tools to contextualize the maturity of the evidence.

**3. Review questions**

1) What types of serious games have been applied in nursing education (platforms, modalities, and formats)?

2) In which nursing education contexts and competencies have serious games been used?

3) How have these interventions been evaluated (outcome domains, instruments, and assessment time points)?

4) What evidence gaps remain across intervention modalities and outcome domains?

4. Eligibility criteria (JBI PCC framework)

**Participants**

Learners in formal nursing education programs, including undergraduate or graduate nursing students and nursing interns/trainees when clearly identified as nursing learners.

**Concept**

Serious game-based teaching and learning interventions with an explicit educational or training purpose. Eligible formats include digital serious games (eg, web-based, mobile, virtual simulation, immersive VR) and offline or hybrid/blended approaches incorporating gameplay and game mechanics.

To distinguish serious games from gamification-only interventions, studies will be classified as serious games when the intervention includes all of the following core features:

A defined goal or endpoint and a rule-based structure.

Interactive tasks with performance feedback linked to learning objectives.

A trackable process (eg, missions, branching decision pathways, multi-round tasks, levels, or progression/unlocking).

Gamification-only interventions that primarily add surface elements (eg, points, badges, or leaderboards) to conventional teaching without game-like task structure or progression will be excluded.

**Context**

Nursing education activities, including theoretical coursework/lectures, skills laboratory training, simulation-based training, and clinical skills learning activities.

**Types of sources**

Primary empirical studies (quantitative, qualitative, or mixed methods), published as full-text journal articles in English or Chinese.

**Exclusion criteria**

Secondary evidence syntheses (eg, systematic reviews, scoping reviews, meta-analyses), non-empirical publications (eg, protocols, guidelines, expert opinions, editorials/commentaries, letters, policy documents), studies not meeting PCC definitions, duplicate reports of the same study (the most complete version retained), and studies for which the full text is not available.

**5. Methods**

**5.1 Protocol and registration**

A protocol was developed a priori to guide eligibility criteria, searching, screening, data charting, and critical appraisal. The protocol was not prospectively registered on an external platform; the final protocol is provided as a supplementary file with the revision.

**5.2 Information sources**

English-language databases: PubMed, Web of Science, Embase, CINAHL, and the Cochrane Library.

Chinese-language databases: China Biology Medicine (CBM), Wanfang Data, China National Knowledge Infrastructure (CNKI), and VIP.

Reference lists of included studies will be screened. Citation tracking may be conducted where feasible.

**5.3 Search strategy**

Search terms were developed using controlled vocabulary and free-text keywords related to serious games and nursing education. The search was run from database inception to January 15, 2026. Full database-specific search strategies are provided in the manuscript appendix.

**5.4 Selection of sources of evidence**

Records were deduplicated before screening. Two reviewers independently screened titles and abstracts, followed by full-text eligibility assessment. Prior to formal screening, the reviewers piloted the eligibility criteria on a sample of records to calibrate interpretation and reduce inconsistency. Disagreements were resolved through discussion, with adjudication by a third reviewer when required. Reasons for full-text exclusion will be documented.

**5.5 Data charting process**

Data were charted using a standardized extraction form piloted on a subset of included studies and refined iteratively. Two reviewers independently extracted data, cross-checked entries, and resolved discrepancies by consensus or third-party adjudication.

**5.6 Data items**

Bibliographic information: first author, publication year, and country/region.

Study design and setting.

Participant characteristics: learner type and sample size, including intervention and comparator group sizes when applicable.

Educational context and target competency domain.

Intervention characteristics: modality/platform, delivery format, and key components.

Key game design features: scenario-based decision points, feedback type and timing, reward structures, progression/unlocking, challenge adaptation, and collaborative/team-based elements when reported.

Intervention duration and intensity (sessions, weeks, and time per session where available).

Comparator, when applicable.

User experience and implementation indicators: usability, acceptability, satisfaction, feasibility indicators, and objective usage/process data (eg, frequency of use, time spent, completion/progression) where reported.

Outcomes: outcome domains, measurement methods/instruments, and assessment time points.

Critical appraisal results using JBI tools, including item-level judgments and an overall summary.

**5.7 Critical appraisal**

Critical appraisal of individual sources of evidence will be conducted using Joanna Briggs Institute critical appraisal tools, selected according to study design. Two reviewers will independently appraise each included study after full-text inclusion. Discrepancies will be resolved through discussion, with adjudication by a third reviewer when necessary. Studies will not be excluded based on critical appraisal results; instead, appraisal findings will be used to contextualize the maturity of the evidence base and inform interpretation of results and evidence gaps.

For each study, item-level judgments will be recorded as Yes, No, Unclear, or Not applicable following JBI guidance. We will report item-level results and an overall appraisal summary for each study, and describe recurring methodological limitations by study design.

**5.8 Analysis and presentation**

Charted data will be summarized using descriptive statistics and narrative synthesis. Results will be presented in tables and figures, including an evidence gap map that cross-tabulates intervention modalities against outcome domains to visualize evidence clusters and gaps.

Critical appraisal findings will be integrated into the narrative synthesis to contextualize conclusions and highlight where claims are constrained by recurrent limitations. Appraisal results will also be used to prioritize recommendations for future study design, reporting, and evaluation.

**5.9 Amendments**

Any protocol amendments will be described in the final manuscript, with a brief rationale and the stage of the review at which the change was made.

**6. Funding and conflicts of interest**

Funding sources and roles of funders will be reported in the manuscript, if applicable.

All authors declare no conflicts of interest.

**7. Data availability**

Data supporting the findings of this scoping review will be provided within the manuscript and its supplementary files. Where access is restricted, the reason and the procedure for requesting access will be described in the Data Availability section.

**8. Dissemination**

Findings will be disseminated through publication in a peer-reviewed journal and, where appropriate, conference presentations.
