# Supplementary material for: Serious Games in Nursing Education: Scoping Review of Applications, Effectiveness, and Future Directions
Source: JMIR Serious Games. 2026 Jun 11;14:e86092. doi: 10.2196/86092 (PMC13258065; doi:10.2196/86092)
Supplement: Multimedia Appendix 2 [file games-v14-e86092-s002.docx]

**List of terms used and search results**

| **No.** | **Database** | **Search No.** | **Terms** | **Search result** |
| --- | --- | --- | --- | --- |
| **1** | **Pubmed (MeSH)** | #1 | ((((Nursing[Title/Abstract] OR Nurses[Title/Abstract] OR nurs* [Title/Abstract]) AND (Education[Title/Abstract] OR Teaching[Title/Abstract] OR educat*[Title/Abstract] OR instruct*[Title/Abstract] OR teach*[Title/Abstract] OR student*[Title/Abstract] OR undergraduate*[Title/Abstract] OR Pupil[Title/Abstract] OR "Student engagement"[Title/Abstract] OR school[Title/Abstract]))) OR ("Students, Nursing"[Mesh])) OR (Education, Nursing[Mesh]) | [216,916](https://pubmed.ncbi.nlm.nih.gov/?term=((((Nursing%5bTitle/Abstract%5d+OR+Nurses%5bTitle/Abstract%5d+OR+nurs*+%5bTitle/Abstract%5d)+AND+(Education%5bTitle/Abstract%5d+OR+Teaching%5bTitle/Abstract%5d+OR+educat*%5bTitle/Abstract%5d+OR+instruct*%5bTitle/Abstract%5d+OR+teach*%5bTitle/Abstract%5d+OR+student*%5bTitle/Abstract%5d+OR+undergraduate*%5bTitle/Abstract%5d+OR+Pupil%5bTitle/Abstract%5d+OR+) |
|  |  | #2 | ((game-based learning[Title/Abstract]) OR ((online[Title/Abstract] OR electronic[Title/Abstract] OR digital[Title/Abstract] OR computer[Title/Abstract] OR virtual[Title/Abstract] OR "mobile application*"[Title/Abstract] OR "mobile app"[Title/Abstract] OR serious[Title/Abstract] OR applied[Title/Abstract] OR "virtual reality"[Title/Abstract] OR "augmented reality"[Title/Abstract] OR "computer based"[Title/Abstract] OR "computer application"[Title/Abstract] OR mobile[Title/Abstract] OR "web based"[Title/Abstract] OR internet[Title/Abstract] OR video[Title/Abstract] OR Educational[Title/Abstract] OR television[Title/Abstract]) AND (game[Title/Abstract] OR games[Title/Abstract] OR gamification[Title/Abstract] OR gaming[Title/Abstract] OR "game-based"[Title/Abstract] OR gamified[Title/Abstract] OR gamifying[Title/Abstract]))) | [27,090](https://pubmed.ncbi.nlm.nih.gov/?term=((game-based+learning%5bTitle/Abstract%5d)+OR+((online%5bTitle/Abstract%5d+OR+electronic%5bTitle/Abstract%5d+OR+digital%5bTitle/Abstract%5d+OR+computer%5bTitle/Abstract%5d+OR+virtual%5bTitle/Abstract%5d+OR+) |
|  |  | #3 | #1 AND #2 | **[777](https://pubmed.ncbi.nlm.nih.gov/?term=(((game-based+learning%5bTitle/Abstract%5d)+OR+((online%5bTitle/Abstract%5d+OR+electronic%5bTitle/Abstract%5d+OR+digital%5bTitle/Abstract%5d+OR+computer%5bTitle/Abstract%5d+OR+virtual%5bTitle/Abstract%5d+OR+)** |
| **2** | **Web of Science** | #1 | (Nursing OR Nurses OR nurs* ) AND (Education OR Teaching OR educat* OR instruct* OR teach* OR student* OR undergraduate* OR Pupil OR "Student engagement" OR school) | 133,339 |
|  |  | #2 | TS=(((online OR electronic OR digital OR computer OR virtual OR "mobile application*" OR "mobile app" OR serious OR applied OR "virtual reality" OR "augmented reality" OR "computer based" OR "computer application" OR mobile OR "web based" OR internet OR video OR Educational OR television) AND (game OR games OR gamiﬁcation OR gaming OR "game-based" OR gamiﬁed OR gamifying))) | 144,125 |
|  |  | #6 | #1 AND #2 | **1046** |
| **3** | **Cochrane** | #1 | MeSH descriptor: [Students, Nursing] this term only | 4914 |
|  |  | #2 | MeSH descriptor: [Education, Nursing] this term only | 15,711 |
|  |  | #3 | (Nursing OR Nurses OR nurs*):ti,ab,kw | 66,776 |
|  |  | #4 | (Education OR Teaching OR educat* OR instruct* OR teach* OR student* OR undergraduate* OR Pupil OR "Student engagement" OR school):ti,ab,kw | 356,955 |
|  |  | #5 | #1 OR #2 | 17,493 |
|  |  | #6 | #3 AND #4 | 26,610 |
|  |  | #7 | #5 OR #6 | 31,501 |
|  |  | #8 | MeSH descriptor: [Mobile Applications] this term only | 4,479 |
|  |  | #9 | MeSH descriptor: [Video Games] this term only | 2,711 |
|  |  | #10 | MeSH descriptor: [Virtual Reality] this term only | 9,881 |
|  |  | #11 | (game-based):ti,ab,kw | 877 |
|  |  | #12 | #8 OR #9 OR #10 OR #11 | 16,986 |
|  |  | #13 | (online OR electronic OR digital OR computer OR virtual OR "mobile application*" OR "mobile app" OR serious OR applied OR "virtual reality" OR "augmented reality" OR "computer based" OR "computer application" OR mobile OR "web based" OR internet OR video OR Educational OR television):ti,ab,kw | 352,890 |
|  |  | #14 | (game OR games OR gamiﬁcation OR gaming OR "game-based" OR gamiﬁed OR gamifying):ti,ab,kw | 12117 |
|  |  | #15 | #13 AND #14 | 6,786 |
|  |  | #16 | #12AND #15 | **877** |
| **4** | **CINAHL** | #1 | ("Students, Nursing+") OR "Education, Nursing+" OR TI ( ((Nursing OR Nurses OR nurs* ) AND (Education OR Teaching OR educat* OR instruct* OR teach* OR student* OR undergraduate* OR Pupil OR "Student engagement" OR school)) ) OR AB ( ((Nursing OR Nurses OR nurs* ) AND (Education OR Teaching OR educat* OR instruct* OR teach* OR student* OR undergraduate* OR Pupil OR "Student engagement" OR school))) | 162,942 |
|  |  | #2 | (Mobile Applications) OR (Video Games) OR (Virtual Reality) OR (Augmented Reality) | 36,439 |
|  |  | #3 | "TI game based learning" OR AB "game based learning" | 828 |
|  |  | #4 | #2 AND #3 | 30,568 |
|  |  | #9 | #1 AND #4 | **1756** |
| **5** | **EMBASE** | #1 | 'nursing student'/exp OR 'nursing education'/exp OR (('nursing'/exp OR 'nursing':ti,ab OR 'nurse':ti,ab OR 'nurses':ti,ab) AND ('education' OR 'teaching' OR 'educate' OR 'instruction' OR 'teach' OR 'student' OR 'undergraduate student' OR 'undergraduate education' OR 'undergraduate' OR 'pupil' OR 'student engagement' OR 'school')) | 374,114 |
|  |  | #2 | 'mobile application'/exp OR 'video game'/exp OR 'virtual reality'/exp OR 'augmented reality'/exp OR 'game-based learning' OR (('online' OR 'electronic' OR 'digital' OR 'computer' OR 'virtual' OR 'mobile application' OR 'mobile app' OR 'serious' OR 'applied' OR 'virtual reality' OR 'augmented reality' OR 'computer based' OR 'computer application' OR 'mobile' OR 'web based' OR 'internet' OR 'video' OR 'educational' OR 'television') AND ('game' OR 'games' OR 'gamiﬁcation' OR 'gaming' OR 'game-based' OR 'gamiﬁed' OR 'gamifying')) | 111,743 |
|  |  | #3 | #1 AND #2 | **745** |
| **6** | **Wanfang Database（Chinese database）** | #1 | 主题:(护理学生 or 护理本科生 or 护理研究生 or 护生 or 护士) | 4,229,166 |
|  |  | #2 | 主题:(护理教育 or 护理教学 or 护理训练) | 162,341 |
|  |  | #3 | 主题:(严肃游戏 or 教育游戏 or 游戏课堂 or 游戏课程 or 游戏教育 or 游戏指导 or 游戏学习 or 游戏模拟 or 游戏培训 or 游戏辅导 or 游戏平台 or 游戏) | 1,938,290 |
|  |  | #4 | #1 AND #2 AND #3 | **709** |
| **7** | **CNKI（Chinese database）** | #1 | 主题: (护理学生 + 护理本科生 + 护理研究生 + 护生 + 护士) | 489,153 |
|  |  | #2 | 主题:(护理教育 + 护理教学 + 护理训练) | 67,875 |
|  |  | #3 | 主题：(严肃游戏 + 教育游戏 + 游戏课堂 + 游戏课程 + 游戏教育 + 游戏指导 + 游戏学习 + 游戏模拟 + 游戏培训 + 游戏辅导 + 游戏平台 + 游戏) | 544,962 |
|  |  | #4 | #1 AND #2 AND #3 | 72 |
| **8** | **CBM（Chinese database）** | #1 | 主题: (护理学生 or 护理本科生 or 护理研究生 or 护生 or 护士) | 246,966 |
|  |  | #2 | 主题:(护理教育 or 护理教学 or 护理训练) | 56,720 |
|  |  | #3 | 主题：(严肃游戏 or 教育游戏 or 游戏课堂 or 游戏课程 or 游戏教育 or 游戏指导 or 游戏学习 or 游戏模拟 or 游戏培训 or 游戏辅导 or 游戏平台 or 游戏) | 57,090 |
|  |  | #4 | #1 AND #2 AND #3 | **87** |
| **9** | **VIP（Chinese database）** | #1 | 题名或关键词：(护理学生 or 护理本科生 or 护理研究生 or 护生 or 护士) | 135015 |
|  |  | #2 | 题名或关键词：(护理教育 or 护理教学 or 护理训练) | 71027 |
|  |  | #3 | 题名或关键词：(严肃游戏 + 教育游戏 + 游戏课堂 + 游戏课程 + 游戏教育 + 游戏指导 + 游戏学习 + 游戏模拟 + 游戏培训 + 游戏辅导 + 游戏平台 + 游戏) | 225430 |
|  |  | #4 | #1 AND #2 AND #3 | **18** |
